# Prediction of Periodontal Disease Progression During Supportive Periodontal Therapy Using an Oral Fluid Lactate Dehydrogenase Activity-Based Test Strip

**DOI:** 10.3390/jcm14248810

**Published:** 2025-12-12

**Authors:** Tatsuo Yamamoto, Satsuki Sato, Yohei Kamata, Takahisa Hirata, Keiichiro Nanashima, Koichiro Irie, Motohiro Komaki

**Affiliations:** 1 Department of Preventive Dentistry and Dental Public Health, Kanagawa Dental University, 82 Inaoka-Cho, Yokosuka 238-8580, Kanagawa, Japan; nanashima@kdu.ac.jp; 2 Department of Advanced Periodontology, Yokohama Clinic, Kanagawa Dental University, 3-31-6 Tsuruya-Cho, Yokohama 221-0835, Kanagawa, Japan; sato.satsuki@kdu.ac.jp (S.S.); ka-mata@kdu.ac.jp (Y.K.); 3 Department of Periodontology, Kanagawa Dental University, 82 Inaoka-Cho, Yokosuka 238-8580, Kanagawa, Japan; t.hirata@kdu.ac.jp (T.H.); m.komaki@kdu.ac.jp (M.K.); 4 Department of Oral Health, Medical and Dental Sciences, Graduate School of Biomedical Sciences, Nagasaki University, 1-12-4 Sakamoto, Nagasaki 852-8523, Nagasaki, Japan; iriko@nagasaki-u.ac.jp

**Keywords:** periodontal diseases, lactate dehydrogenase, oral fluid, progression, supportive periodontal therapy, cohort studies

## Abstract

**Background/Objectives**: Periodontal disease may progress despite supportive periodontal therapy (SPT), and conventional clinical parameters exhibit limited predictive value. This study evaluated the prognostic utility of measuring the oral fluid lactate dehydrogenase (LD) activity using a chairside test strip to predict disease progression during SPT. **Methods**: A cohort of 92 patients (33 males and 59 females; median age, 68 years) undergoing SPT at a university clinic in 2023 were followed up for approximately one year. Oral fluid LD activity was measured using a test strip, and clinical periodontal parameters were assessed at baseline and follow-up. Periodontitis progression was defined as ≥4 sites showing a 2 mm increase in the probing pocket depth, which reached ≥4 mm. Receiver operating characteristic analyses and stepwise logistic regression were used to evaluate the predictive performance of oral fluid LD activity and develop a multivariate model. **Results**: Seventeen patients (18.5%) developed progressive periodontitis. Progressive cases demonstrated significantly higher baseline LD activity and periodontal parameters. The optimal cutoff value for LD activity was 3.5, yielding a sensitivity, specificity, and negative predictive value of 1.00, 0.53, and 1.00, respectively. Logistic regression identified oral fluid LD and medications as independent predictors, with the multivariate model achieving an area under the curve of 0.785. **Conclusions**: Oral fluid LD activity measured using the test strip provided prognostic information for periodontitis progression during SPT. Incorporating LD with information obtained from medical interviews did not markedly enhance the predictive accuracy. This rapid and noninvasive method may facilitate risk-based patient management and personalized supportive care.

## 1. Introduction

Periodontal disease is a highly prevalent chronic inflammatory disorder characterized by the progressive destruction of periodontal tissues and eventual tooth loss [1]. Contemporary global estimates indicate that the prevalence, incidence, and disability-adjusted life years attributable to periodontitis increased from 1990 to 2019, with a disproportionate burden in socioeconomically disadvantaged regions, underscoring its public health importance [2]. Beyond local sequelae, periodontitis is bidirectionally associated with multiple systemic conditions, notably diabetes mellitus and cardiovascular diseases, suggesting that effective periodontal management may contribute to broader health outcomes [3,4,5]. Given the chronic, relapsing nature of periodontitis, the accurate prediction of disease progression is essential for timely and individualized interventions during the maintenance phase.

Supportive periodontal therapy (SPT), also termed supportive periodontal care (SPC), is an integral guideline-endorsed component of the treatment pathway for periodontitis that aims to maintain periodontal stability after active therapy and prevent recurrence [6,7]. Nevertheless, a subset of patients exhibits recurrence or continued breakdown during SPT. Cohort studies and systematic reviews indicate that tooth loss during long-term maintenance is generally low but non-negligible and that adherence to SPT is a key determinant of outcomes [8]. Meta-analytic data further shows that noncompliance during SPT increases the risk of tooth loss relative to compliant patients, highlighting the need for robust and practical risk stratification tools to guide recall intervals and adjunctive measures [9].

Conventional risk assessment approaches rely heavily on clinical parameters, including the probing pocket depth (PPD), clinical attachment level (CAL), bleeding on probing (BOP), and radiographic bone loss [10]. However, their ability to capture imminent risks is limited because they predominantly reflect the current or historical tissue status rather than subclinical activity [6]. Notably, the presence of BOP has been linked to future breakdown risk, while its absence is a strong indicator of site-level periodontal stability; however, BOP alone is insufficient for comprehensive risk prediction at the patient level [11]. Moreover, most clinical examinations are invasive [12] and operator-dependent [13]. These constraints motivate the integration of biologically responsive indicators that reflect ongoing tissue-destructive processes.

Oral fluid biomarkers offer a noninvasive, chairside-accessible window into host responses and tissue degradation. Recent reviews and translational studies have emphasized the potential of oral fluid-based diagnostics for case detection and activity monitoring in periodontal diseases [14]. Among candidate markers, lactate dehydrogenase (LD), an intracellular enzyme released upon cell damage and necrosis, has emerged as a plausible indicator of active periodontal tissue breakdown [15]. Chairside-compatible LD activity tests have been developed and validated analytically, supporting their feasibility for routine practice [16,17].

More recently, cross-sectional clinical investigations employing commercially available LD test kits have demonstrated significant correlations between oral fluid LD activity and BOP rates, PPD distributions, and the periodontal inflamed surface area (PISA), including a Japanese-specific PISA algorithm, thereby linking LD activity to a quantifiable inflammatory burden [18]. Multicenter data also suggest clinically actionable cutoffs (e.g., LD ≥ 4 on a 1–10 kit scale) for screening periodontal disease, with sensitivity/specificity profiles supportive of point-of-care use [19]. Nevertheless, despite growing evidence of its feasibility, the predictive validity of oral fluid LD activity for future periodontal disease progression during SPT, especially in direct comparison with conventional clinical parameters, remains insufficiently established. While earlier studies on oral fluid biomarkers have explored progression prediction, prospective cohort-level validation in the context of SPT and strategies to enhance the predictive accuracy are still needed.

Accordingly, this cohort study aimed to evaluate whether baseline oral fluid LD activity, measured using a commercially available test strip, can predict future periodontal disease progression in patients undergoing SPT. The purpose of the oral fluid LD test in this context is not to monitor the therapeutic response to SPT, as LD was measured only once at baseline and no post-SPT measurement was performed. Rather, it serves as a baseline prognostic biomarker that helps identify individuals at increased risk of subsequent breakdown despite receiving regular maintenance care. In addition, we compared the predictive performance of oral fluid LD with that of conventional periodontal parameters. We also explored whether combining oral fluid LD with information obtained from medical interviews could enhance predictive accuracy and support risk-based maintenance protocols.

## 2. Materials and Methods

### 2.1. Study Participants

**Figure 1:**
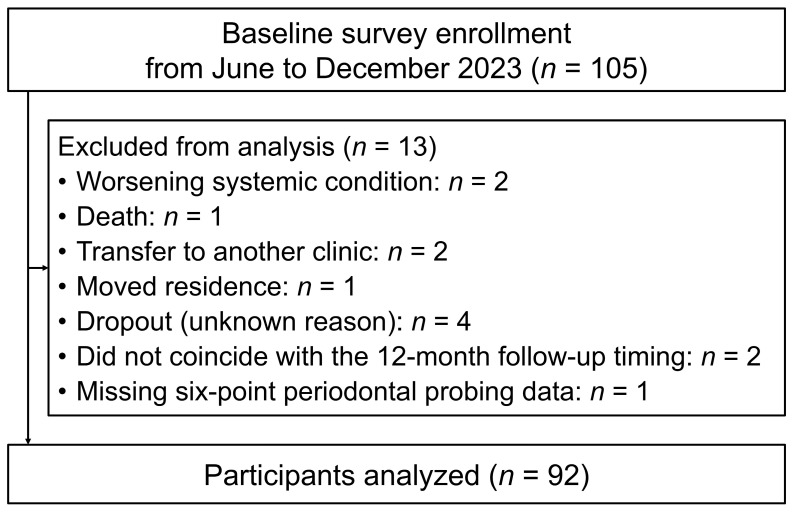
Flow diagram of participant selection.

Of 105 adult patients diagnosed with stages II–IV periodontitis who visited Yokohama Clinic, Kanagawa Dental University, Japan, between June and December 2023 to receive SPT, 92 (33 males and 59 females) were included in the analysis (Figure 1). These individuals returned for follow-up approximately one year, at which time all data required for the study were successfully collected. The baseline patient age range was 34–92 years (median: 68 years; interquartile range: 55–75.8 years). The exclusion criteria at baseline were as follows: age < 20 years, fewer than five teeth, current smokers, use of antibiotics within the past 3 months, stomatitis, oral wounds, or acute inflammation on the day of the oral examination.

### 2.2. Data Collection Using Medical Records and Questionnaires

Age and sex data were obtained from medical records; data on the smoking history, medications taken, and diseases being treated were collected by questioning the participants.

### 2.3. Measurement of LD Activity in Oral Fluid

Oral fluid LD activity was measured using a test strip (LDH Test NAGATA; Nagata Sangyo Co., Shiso, Japan), which contained 3.347 mg/mL nicotinamide adenine dinucleotide, 500 U/mL diaphorase, 5.0 mg/mL nitroblue tetrazolium, 12 mg/mL Tris buffer, 40 mg/mL lithium lactate, and 10 mg/mL bovine serum albumin. In the presence of LD, formazan (purple) was produced from nitroblue tetrazolium (faint yellow) [16,17]. Resting oral fluid was collected without stimulation. Participants first rinsed their mouth briefly with water to remove debris and expelled it completely. They then remained still for approximately 1 min to allow oral fluid to accumulate naturally. Subsequently, about 0.5 mL of oral fluid was obtained by passive drooling into a disposable paper cup. The LD test strip was then applied directly to the pooled oral fluid in the cup according to the manufacturer’s protocol. The resulting color change indicated LD activity. The LD activity was recorded after 60 s according to the scale guide (Lot CC2; scale, 1–10). All examiners were calibrated by the same experienced dentist (K.I.), and the corresponding color value was determined. Flow rate of oral fluid (mL/min) was not measured, as this parameter is not required for interpreting the LD test strip.

### 2.4. Oral Examination

After measuring the oral fluid LD activity, two trained dentists (S.S. and K.Y.) examined the participants’ oral health status. They counted the number of teeth present and measured the PPD and BOP at six sites per tooth for all teeth using a manual probe (PCP-UNC 15; Hu-Friedy, Chicago, IL, USA).

The same oral examination was performed approximately one year after baseline (median: 12 months; interquartile range: 11–13 months).

### 2.5. Calculation of PISA and PISA-Japanese Version

PISA was calculated using the method proposed by Nesse et al. [20], which converts site-level PPD and BOP measurements into a continuous estimate of the PISA. The Japanese version (PISA-Japanese) applies a modified tooth-type–specific root surface area algorithm validated for Japanese dentitions [21]. Both indices were computed using full-mouth measurements, and descriptive comparisons were reported [21].

### 2.6. Ethical Issues

Written informed consent for all data used in the analysis was obtained from all participants in accordance with the Ethical Guidelines for Medical and Biological Research Involving Human Subjects. The study protocol was approved by the Ethics Committee of Kanagawa Dental University (approval no. 967; 8 November 2023). The study was conducted in accordance with the principles of the Declaration of Helsinki.

### 2.7. Statistical Analyses

Statistical power analysis for the receiver operating characteristic (ROC) curves was conducted using the “power.roc.test” function in the pROC package of R (version 4.3.1). Using an expected area under the curve (AUC), significance level, and desired power of 0.80, 0.05, and 0.90, respectively, the minimum required sample size was estimated to be nine cases and 35 controls for a one-sided test. The ratio of controls to cases (kappa) was set to 4.0.

Periodontitis progression was defined as having at least four sites with a ≥2 mm increase in PPD, yielding a PPD of at least 4 mm at follow-up [22]. Baseline characteristics and clinical parameters at baseline and follow-up were compared between patients with and without progression using Fisher’s exact test or the Mann–Whitney *U* test.

ROC curves were constructed to set the cutoff points for oral fluid LD activity and periodontal parameters, and the points showing the highest sum of sensitivity and specificity were selected for the progression of periodontitis. Positive predictive values (PPVs), negative predictive values (NPVs), and likelihood ratios were calculated for oral fluid LD activity and periodontal parameters.

To improve the accuracy of periodontitis progression prediction based on oral fluid LD activity, we performed stepwise logistic regression analysis (entry/removal criteria: *p* < 0.05/*p* > 0.10) using the presence or absence of periodontitis progression as the dependent variable and oral fluid LD activity and variables that exhibited significant differences between the progression and non-progression groups based on data other than the baseline periodontal tissue examination as explanatory variables. Finally, a formula was derived to calculate the risk of periodontitis progression. The model discrimination was evaluated using the AUC in the ROC analysis. Additionally, the prediction performance of the model was evaluated using the sensitivity, specificity, PPV, NPV, and likelihood ratio.

All analyses, except for the statistical power analysis of the ROC curve, were performed using IBM SPSS Statistics (version 29.0; SPSS Japan Inc., Tokyo, Japan).

## 3. Results

Table 1 summarizes the comparison of baseline clinical characteristics between patients with and without periodontal progression. Of the 92 patients, 17 (18.5%) were classified as progressive. The progression group exhibited a significantly higher proportion of male participants and a higher prevalence of hypertension and medication use than the non-progression group (*p* < 0.05). Baseline oral fluid LD activity and all periodontal parameters were significantly higher in the progression group than in the non-progression group (*p* < 0.05).

Table 2 summarizes the comparisons of follow-up clinical characteristics between patients with and without periodontal progression. All periodontal parameters and the number of sites with periodontal progression were significantly higher in the progression group than in the non-progression group (*p* < 0.01).

**Figure 2:**
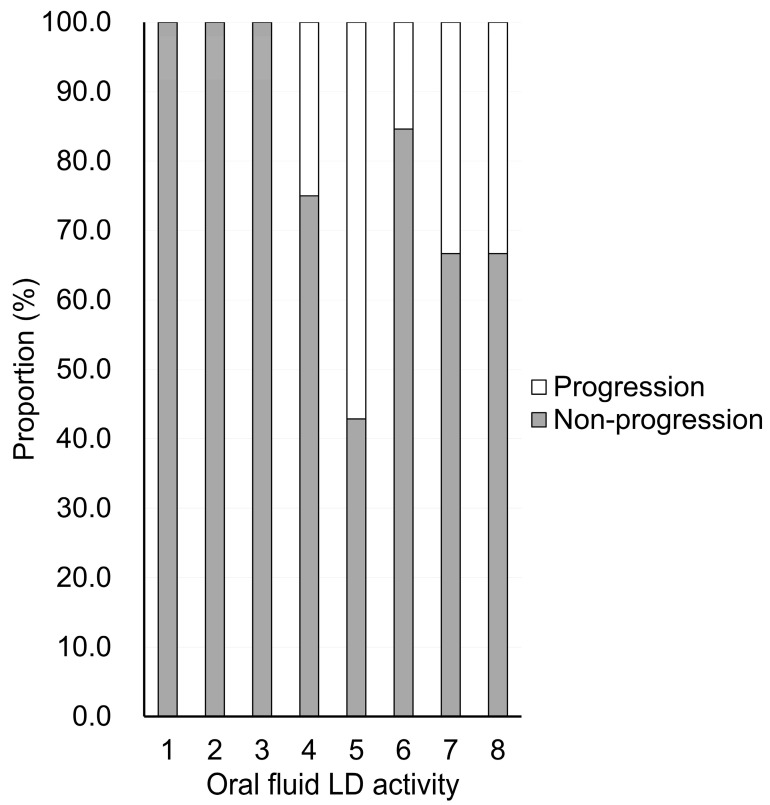
Proportion of patients with and without progression at each baseline oral fluid LD activity.

Figure 2 shows the proportion of patients with and without progression at each baseline oral fluid LD activity. None of the patients with oral fluid LD activity of 1–3 showed periodontitis progression. The proportion of patients with oral fluid LD activity of ≥4 who had periodontal progression ranged from 15.4 to 57.1%.

Table 3 presents the diagnostic values of oral fluid LD activity and periodontal parameters. The AUCs for oral fluid LD activity and periodontal parameters were 0.76 and 0.75–0.83, respectively. The cutoff value for oral fluid LD activity was 3.5, with a sensitivity, specificity, PPV, NPV, and likelihood ratio of 1.00, 0.53, 0.33, 1.00, and 2.14, respectively. The sensitivity and specificity of the periodontal parameters’ ranges were 0.65–1.00 and 0.56–0.85, respectively.

Table 4 displays the results of the logistic regression analyses with stepwise variable selection for periodontitis progression. Oral fluid LD activity and medication use independently predicted periodontitis progression. A multivariate logistic regression predictive model was developed based on a logistic regression equation, to determine the risk of periodontitis progression, as follows:

*p* = (exp (0.548 × oral fluid LD activity + 1.236 × (medication use: yes = 1, no = 0) − 4.542))/(1 + (exp (0.548 × oral fluid LD activity + 1.236 × (medication use: yes = 1, no = 0) − 4.542)))

The AUC (95% confidence interval), optimal threshold cutoff value (determined by the highest Youden index value), sensitivity, specificity, PPV, NPV, and likelihood ratio were 0.785 (0.689–0.881), 0.1202, 1.00, 0.55, 0.33, 1.00, and 2.21, respectively.

## 4. Discussion

This cohort study demonstrated that baseline oral fluid LD activity measured using a chairside test strip was significantly associated with the risk of subsequent periodontal disease progression during SPT. It should be noted that the present study was not intended to evaluate the effectiveness of SPT itself. If our aim had been to assess treatment response, oral fluid LD activity would have required repeated measurements before and after SPT. Instead, oral fluid LD was measured only at baseline, consistent with a prognostic design evaluating the risk of subsequent progression rather than treatment-induced changes. To our knowledge, this is the first longitudinal study to validate the prognostic utility of oral fluid LD activity for predicting periodontitis progression in patients receiving SPT.

Previous cross-sectional studies have shown significant associations between oral fluid LD activity measured using a test strip and periodontal disease severity [16,17,18,19]; however, evidence for its predictive validity has been limited. The present findings expand our knowledge by demonstrating that oral fluid LD activity not only reflect current periodontal inflammation but also have prognostic value when assessed over time. This is clinically meaningful, as it suggests that oral fluid LD testing can move beyond screening and toward risk-based patient management in SPT.

The cutoff value for oral fluid LD activity used in this study to distinguish the presence or absence of periodontal disease progression, as determined using test strips, was similar to that used in previous studies where oral fluid samples underwent laboratory measurements [23]. The cutoff value in this study was 3.5, which corresponds to 0.31 U/mL, while that in the previous study was 0.262 U/mL.

Oral fluid LD testing has several diagnostic advantages, as it is rapid, noninvasive, and can be performed chairside within 1 min; in contrast, the measurement of conventional periodontal parameters, such as the PPD and BOP, is invasive [12] and operator-dependent [13]. Importantly, the high NPV (1.00) observed suggests that this test may be particularly useful for identifying low-risk patients who require fewer frequent recall visits. Notably, this testing strip demonstrated no progression of periodontal disease in patients with baseline oral fluid LD activity of ≤3. Conversely, those with elevated oral fluid LD values should be prioritized for closer monitoring and tailored preventive strategies, thereby contributing to a more efficient allocation of clinical resources.

The biological rationale for these findings is that LD is released during cell and tissue damage, reflecting periodontal breakdown and inflammation. Thus, elevated oral fluid LD activity is likely to capture ongoing tissue destruction, which explains their association with future disease progression. In particular, the diagnostic value of oral fluid LD activity was similar to that of BOP (Table 3). These results are consistent with those of previous cross-sectional studies that showed a correlation between oral fluid LD activity and BOP [16], suggesting that oral fluid LD activity may reflect the same degree of periodontal tissue destruction as BOP.

In this study, we attempted to improve the screening accuracy using a multivariate logistic regression model that included information easily obtained through questionnaires, in addition to oral fluid LD activity; however, this did not result in improved accuracy. Oral fluid LD likely reflects host factors, which may be related to the lack of evaluation of microbial factors. Including microbial factors may improve the screening accuracy [23]. Additionally, combining oral fluid LD activity with activity of other oral fluid biomarkers, microbial profiles, or machine learning-based risk models [24] may further improve the diagnostic performance. Ultimately, the integration of oral fluid LD testing into routine SPT could provide a cost-effective, noninvasive tool to support personalized periodontal care, enabling clinicians to better identify at-risk patients and optimize treatment strategies.

Logistic regression analysis revealed that medication use was independently and significantly associated with periodontitis progression, independent of oral fluid LD activity. This is because patients receiving medication tend to have a high prevalence of diabetes and hypertension, male sex, and older age. Previous studies have indicated that these factors increase the risk of periodontal disease progression and tooth loss during the SPT period [8,25].

This study had several strengths, including its longitudinal design, standardized clinical assessment, and use of a validated diagnostic kit. Nevertheless, several methodological limitations should be acknowledged. First, disease progression in this study was defined based on changes in PPD, because CAL measurements were not consistently obtainable at all sites during routine SPT visits in our clinical setting. Although CAL is widely regarded as the gold standard for evaluating periodontitis progression [26,27], the unavailability of standardized CAL records required reliance on PPD-based criteria. A previous longitudinal study used CAL-based definitions and reported cutoff values for LD activity comparable to those observed in the present study [23], supporting the general validity of our findings; however, the lack of CAL data in the present study may have resulted in misclassification in some cases.

Furthermore, baseline severity of periodontal disease and inflammatory burden—both of which can substantially influence oral fluid LD activity—were not fully controlled in our analyses. Oral fluid LD is inherently a biological indicator of tissue breakdown and host inflammatory response, and thus it naturally correlates with baseline periodontal parameters such as PPD, BOP, and PISA. Importantly, oral fluid LD represents a baseline biological marker of inflammatory burden and tissue breakdown. Because periodontal parameters (PPD, BOP, and PISA) lie on the same causal pathway, adjusting for them would remove relevant prognostic information and introduce overadjustment bias. For this reason, oral fluid LD was evaluated as an independent baseline predictor.

Second, the follow-up period of approximately one year is relatively short for a chronic and intermittently active condition such as periodontitis. Although significant associations between baseline oral fluid LD activity and progression were observed, a one-year observation window may be insufficient to fully capture long-term disease trajectories or to determine whether LD activity can reliably predict multi-year changes in clinical attachment or tooth loss. Therefore, the long-term prognostic value of oral fluid LD activity remains to be established, and future multi-year cohort studies are warranted.

Third, although several systemic and behavioral variables were collected, important confounders—including systemic inflammatory conditions, genetic susceptibility, and detailed oral hygiene behaviors [28]—were not measured. These unassessed factors may influence both periodontal status and oral fluid LD activity, potentially affecting the observed associations. Incorporating systemic biomarkers, genetic profiling, or high-resolution assessments of oral hygiene in future studies will be necessary to clarify the independent contribution of oral fluid LD to the risk of periodontitis progression.

Fourth, although systemic conditions and medication use may affect oral fluid biomarkers, additional analyses in this study showed no meaningful differences in oral fluid LD activity according to hypertension or medication status, and LD remained independently associated with disease progression after adjustment. Nonetheless, unmeasured systemic influences cannot be completely excluded.

Fifth, current smokers were excluded, and only a small proportion of participants had diabetes mellitus. Because both smoking and diabetes can alter LD metabolism [29,30] and are strongly associated with periodontal disease severity [8,25], the limited representation of these groups restricts the generalizability of the findings. Whether the LD-based test strip maintains predictive performance in populations with higher metabolic or smoking-related risk remains uncertain. Validation of this approach in broader and more heterogeneous patient cohorts is therefore required.

Finally, as the test strip requires only a small amount of non-stimulated oral fluid, flow rate of oral fluid was not measured in this study. Because oral fluid LD originates primarily from periodontal tissue breakdown rather than salivary gland secretion, its dependency on flow of oral fluid is considered limited. Nevertheless, future studies incorporating flow-rate assessment may help refine the interpretation of oral fluid LD activity.

The LD-based test is not intended to replace clinical judgment or to determine whether surgical therapy is preferable to SPT. Rather, LD-based test may support risk stratification during maintenance and help identify patients who may benefit from closer monitoring.

## 5. Conclusions

The oral fluid LD activity measured at baseline using a rapid, noninvasive chairside test strip was meaningfully associated with the likelihood of periodontal disease progression during SPT. The test is therefore best interpreted as a prognostic tool that may help clinicians identify individuals who require closer monitoring or more intensive maintenance, rather than as a measure of treatment response to SPT itself. Future large-scale, multicenter studies with longer follow-up periods are warranted to confirm the prognostic utility of oral fluid LD activity and to explore its integration with other biomarkers or machine learning-based predictive models.

## Figures and Tables

**Table 1:** Comparison of the baseline clinical parameters of patients with and without periodontal progression.

Variable	Non-Progression (*n* = 75)	Progression (*n* = 17)	*p*-Value
Sex			
Male	23 (30.7%)	10 (58.8%)	0.030
Female	52 (69.3%)	7 (41.2%)	
Age (years)	68.0 (55.0–75.0)	69.0 (60.0–76.0)	0.484
Past smoking			
No	70 (93.3%)	15 (88.2%)	0.383
Yes	5 (6.7%)	2 (11.8%)	
Diabetes mellitus			
No	72 (96.0%)	16 (94.1%)	0.565
Yes	3 (4.0%)	1 (5.9%)	
Hypertension			
No	60 (80.0%)	9 (52.9%)	0.026
Yes	15 (20.0%)	8 (47.1%)	
Medication			
No	50 (66.7%)	7 (41.2%)	0.048
Yes	25 (33.3%)	10 (58.8%)	
Oral fluid LD activity	3 (2–5)	5 (5–6)	<0.001
Number of teeth present	26 (21–28)	25 (24–26)	0.808
BOP rate (%)	2.9 (1.3–5.4)	6.0 (4.8–9.1)	<0.001
Mean PPD (mm)	2.1 (1.9–2.3)	2.4 (2.2–2.6)	<0.001
PPD ≥ 4 mm, sites	2 (0–5)	14 (6–20)	<0.001
PPD ≥ 4 mm, rate (%)	1.2 (0.0–3.9)	9.3 (4.8–13.3)	<0.001
PPD ≥ 6 mm, sites	0 (0–1)	3 (1–6)	<0.001
PPD ≥ 6 mm, rate (%)	0.0 (0.0–0.7)	2.0 (0.6–4.1)	<0.001
PISA (mm^2^)	28.6 (17.1–65.3)	113.5 (49.9–176.2)	<0.001
PISA-Japanese (mm^2^)	37.4 (21.6–86.2)	132.5 (70.6–171.0)	<0.001

Data are expressed as numbers (%) or medians (25th–75th percentiles). Fisher’s exact test was performed for categorical variables, and the Mann–Whitney *U* test was conducted for continuous variables. BOP: bleeding on probing, LD: lactate dehydrogenase, PISA: periodontal inflamed surface area, PPD: probing pocket depth.

**Table 2:** Comparison of the follow-up clinical parameters of patients with and without periodontal progression.

Variable	Non-Progression (*n* = 75)	Progression (*n* = 17)	*p*-Value
Number of teeth present	26 (21–28)	25 (24–25)	0.442
BOP rate (%)	1.8 (0.0–5.8)	5.6 (4.7–10.1)	0.002
Mean PPD (mm)	2.1 (1.8–2.3)	2.6 (2.2–2.8)	<0.001
PPD ≥ 4 mm, sites	1 (0–4.5)	17 (10–20)	<0.001
PPD ≥ 4 mm, rate (%)	0.9 (0.0–3.1)	10.7 (7.9–14.7)	<0.001
PPD ≥ 6 mm, sites	0 (0–1)	5 (4–9)	<0.001
PPD ≥ 6 mm, rate (%)	0.0 (0.0–0.6)	3.3 (2.6–6.0)	<0.001
PISA (mm^2^)	18.1 (0.0–71.8)	125.7 (81.5–164.5)	<0.001
PISA-Japanese (mm^2^)	31.0 (0.0–92.8)	129.3 (89.4–177.7)	<0.001
Number of sites with progression	0 (0–1)	6 (5–7)	<0.001

Data are expressed as medians (25th–75th percentiles). The Mann–Whitney *U* test was conducted. BOP: bleeding on probing, PISA: periodontal inflamed surface area, PPD: probing pocket depth.

**Table 3:** Diagnostic values of oral fluid LD activity and periodontal parameters.

Variable	Cutoff Point	Area Under the Curve	Sensitivity	Specificity	Positive Predictive Value	Negative Predictive Value	Likelihood Ratio
Oral fluid LD activity	3.5	0.76	1.00	0.53	0.33	1.00	2.14
BOP rate (%)	3.3	0.78	1.00	0.56	0.34	1.00	2.27
Mean PPD (mm)	2.4	0.77	0.65	0.83	0.46	0.91	3.74
PPD ≥ 4 mm, sites	5.5	0.83	0.76	0.77	0.43	0.94	3.37
PPD ≥ 4 mm, rate (%)	4.7	0.81	0.76	0.80	0.46	0.94	3.83
PPD ≥ 6 mm, sites	1.5	0.77	0.65	0.84	0.48	0.91	4.04
PPD ≥ 6 mm, rate (%)	1.5	0.75	0.65	0.84	0.48	0.91	4.04
PISA (mm^2^)	44.5	0.82	0.94	0.63	0.36	0.98	2.52
PISA-Japanese (mm^2^)	111.1	0.80	0.65	0.85	0.50	0.91	4.40

BOP: bleeding on probing, LD: lactate dehydrogenase, PISA: periodontal inflamed surface area, PPD: probing pocket depth.

**Table 4:** Results of logistic regression analyses with stepwise variable selection for periodontitis progression.

Independent Variables	Coefficients	Standard Error	Wald Value	Odds Ratio	95% Confidence Interval		*p*-Value
					Lower	Upper	
Oral fluid LD activity	0.548	0.182	9.018	1.730	1.21	2.473	0.003
Medication (yes: 1, no: 0)	1.236	0.605	4.181	3.443	1.053	11.261	0.041
Constant	−4.542	1.063	18.266	0.011	-	-	<0.001

Dependent variable: 0 = non-progression, 1 = progression. Sex and hypertension were excluded as independent variables. LD: lactate dehydrogenase.

## Data Availability

The data supporting the findings of this study are available upon request from the corresponding author. The data are not publicly available due to privacy or ethical restrictions.

## Abbreviations

The following abbreviations are used in this manuscript:

AUC	area under the curve
BOP	bleeding on probing
CAL	clinical attachment level
LD	lactate dehydrogenase
NPV	negative predictive value
PISA	periodontal inflamed surface area
PPD	probing pocket depth
PPV	positive predictive value
ROC	receiver operating characteristic
SPC	supportive periodontal care
SPT	supportive periodontal therapy
